# Predicting regional coastal sea level changes with machine learning

**DOI:** 10.1038/s41598-021-87460-z

**Published:** 2021-04-07

**Authors:** Veronica Nieves, Cristina Radin, Gustau Camps-Valls

**Affiliations:** Image Processing Laboratory, University of Valencia, Valencia, Spain

**Keywords:** Computer science, Physical oceanography, Projection and prediction

## Abstract

All ocean basins have been experiencing significant warming and rising sea levels in recent decades. There are, however, important regional differences, resulting from distinct processes at different timescales (temperature-driven changes being a major contributor on multi-year timescales). In view of this complexity, it deems essential to move towards more sophisticated data-driven techniques as well as diagnostic and prognostic prediction models to interpret observations of ocean warming and sea level variations at local or regional sea basins. In this context, we present a machine learning approach that exploits key ocean temperature estimates (as proxies for the regional thermosteric sea level component) to model coastal sea level variability and associated uncertainty across a range of timescales (from months to several years). Our findings also demonstrate the utility of machine learning to estimate the possible tendency of near-future regional sea levels. When compared to actual sea-level records, our models perform particularly well in the coastal areas most influenced by internal climate variability. Yet, the models are widely applicable to evaluate the patterns of rising and falling sea levels across many places around the globe. Thus, our approach is a promising tool to model and anticipate sea level changes in the coming (1–3) years, which is crucial for near-term decision making and strategic planning about coastal protection measures.

## Introduction

Short-term variations in regional coastal sea levels depend on a combination of nearshore and offshore processes, including fast-paced changes like those associated to high tides or storms and interannual to multi-year changes driven by relatively large temperature oscillations (thermosteric variations) in large open ocean areas—just to name a few^1^. More particularly, open ocean, temperature changes down the water column to 700 m depth are still influencing regional coastal sea level variability changes^2,3^, and they are closely tied to internal natural climate variability^4–6^. Internal climate variability due to natural fluctuations of the ocean–atmosphere coupled system (such as the Pacific Decadal Oscillation) can lead to periods when the upper ocean warms more slowly or more rapidly, which has direct implications for sea level change^6^. Nevertheless, climate is a highly complex and networked dynamical system (with no simple relationships) that can change naturally in unexpected ways^7,8^. From this perspective, advanced statistical analyses, including machine learning (ML) methods, can provide useful insight.

In recent years, ML techniques have provided significant advances in the modeling of physical variables at both global and local scales^9–11^. These techniques can help identify complex relationships between explanatory variables, and, at the same time, establish “relevance metrics” (i.e. ranking of the level of importance of each variable). This way, we can optimally combine multiple data towards explainability and prediction of events. However, efforts to develop and implement these techniques in the context of the ocean to forecast short-term changes, including near-future regional sea-level predictions, remain largely unexplored^12–15^.

**Figure 1:**
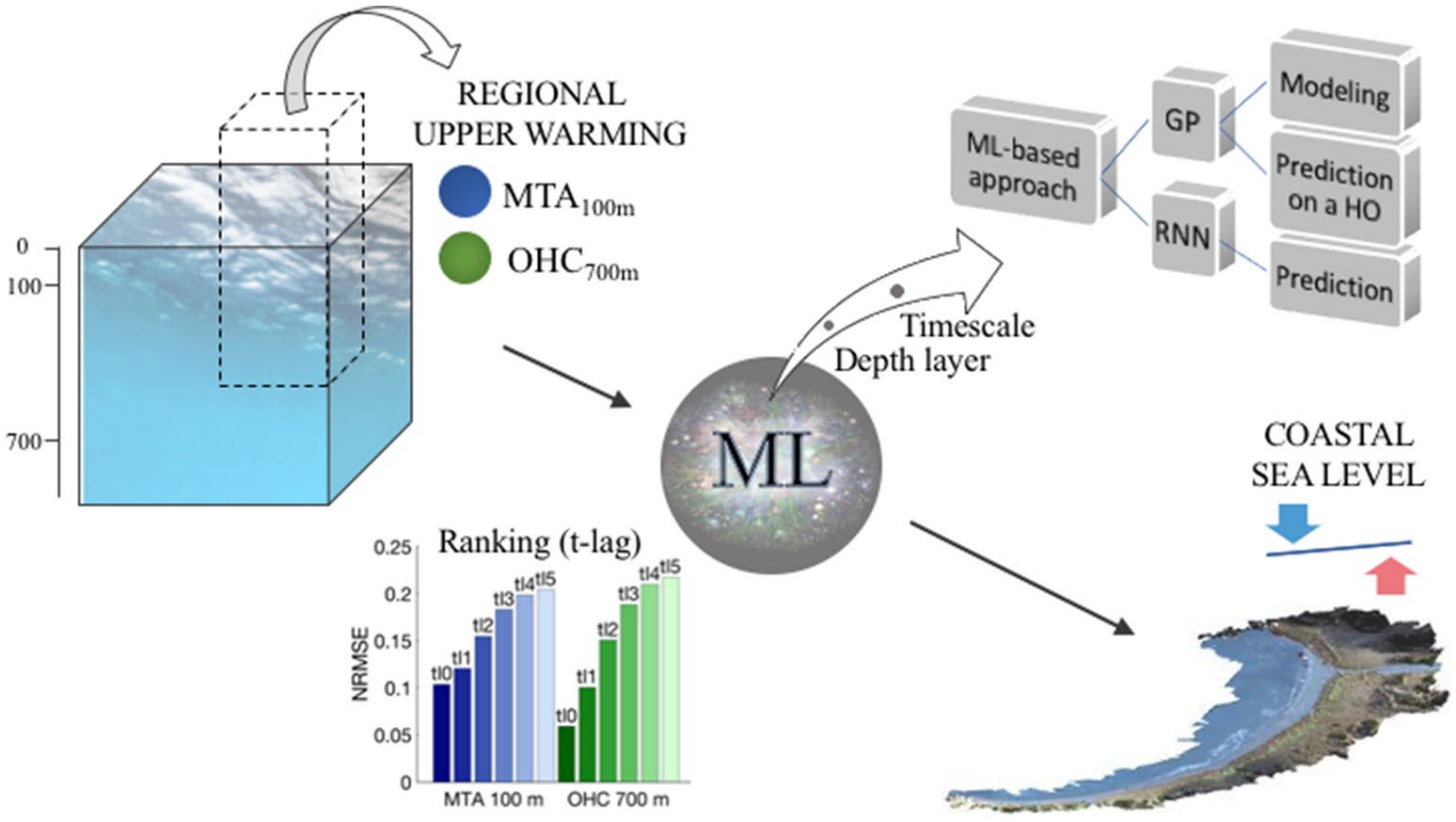
Illustration of our ML approach. The proposed approach combines physical variables (that reflect upper-ocean temperature changes) in open ocean regions and ML techniques (i.e., the nonlinear GP and RNN methods) to assess coastal sea level variability in these regions across a range of timescales. This framework includes a relevance criterion to rank the physical variables according to their performance modeling such changes, while also considering different time lags (Figs. S1-S4).

In this study, we aim to perform a comprehensive evaluation of thermal expansion in open ocean regions driving changes in coastal sea levels to model and pinpoint the “tendency” of increase/decrease in sea level for the next years. Rather than using physics-based equations to describe such changes, our ML models are informed by upper-ocean temperature proxy estimates which, in turn, reflect the underlying physics (e.g., ocean dynamics and internal variability)^3^. The analysis was done by assessing the specific role of the different elements (including different oceanic depth layers and timescales) with nonlinear ML algorithms. Contributions of land movements, changes in tidal range and storm surges to relative sea level were excluded from the analysis. Our innovative approach (Fig. 1) has been tested in 26 coastal regions surrounding the Pacific, Indian, and Atlantic basins. As demonstrated in the case studies, the large temperature fluctuations (by short-term climate changes and ocean circulation) are dominating the patterns of sea level variability changes for most of the regions.

## Results and discussion

### ML helps modeling regional sea level variability changes

**Figure 2:**
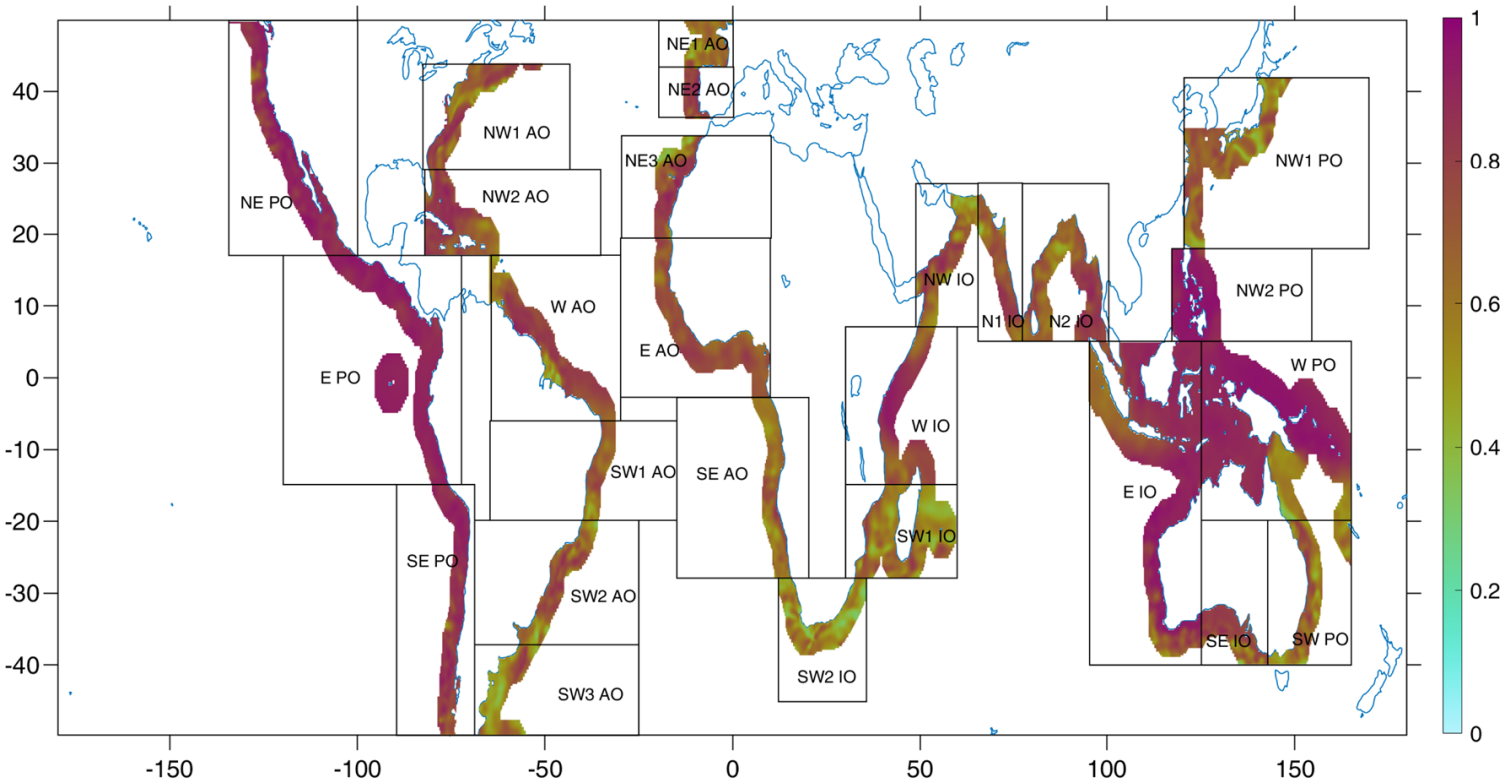
Correlation map between the regional (GP) model and observed sea level estimates at each location of the regions indicated in the boxes and 1993–2018 period. For each specific coastal location, we considered the neighboring data grid points as well, using a weighting local (scaling) function, to account for possible offsets from data^31^. The map was generated using the software MATLAB (version 2019a) http://www.mathworks.com/products/matlab/.

To model sea level variations in the coastal regions from upper-ocean temperature changes in the open ocean (a proxy for climate variability conditions), we first calculate the median estimate within each ocean region (Fig. 2) for each variable (sea level anomaly, SLA; vertically averaged temperature anomaly, MTA; depth-integrated temperature or heat content, OHC) over the period 1993–2018 (see Materials and Methods). Note that the various temperature-based estimates (MTA and OHC) were calculated across different depth layers (i.e. from 0 to 100 and 700 m depth). Since the patterns of both MTA 700 m and OHC 700 m are deemed similar^16,17^, we only show the latter (as well as MTA 100 m) here.

The study domain covers the tropical to mid-latitudes (50°S to 50°N). However, our main goal is to investigate low-latitude sea level changes induced by natural regimes of variability, which operates away from the melting glaciers and other effects^18^. In addition, records have been detrended and smoothed to emphasize interannual to multi-year variability. However, only analyses of data smoothed with a 3-year moving average filter are given here by way of example (see Supplementary Information for results at other timescales). Also point out that we defined relatively wide ocean regions chosen for their significant climate patterns, like the Pacific Decadal Oscillation (PDO), the North Atlantic Oscillation (NAO) or the Indian Ocean Dipole (IOD), or for their physical regimes (i.e., large-scale transport dynamics), such as in the Kuroshio‐Oyashio Extension or the Agulhas Current (Fig. 2). Similar results were observed considering slightly different regions.

Our first target was to use Gaussian Processes (GPs) to determine spatiotemporal relationships between the temperature estimates (MTA and OHC) in the open sea regions and the coastal SLA estimates of these regions, as our intention was to also highlight the influence of the open ocean on the coast^19^. GPs are versatile ML methods for non-parametric nonlinear regression with prediction uncertainty^20^, which typically yields good accuracy results in the geosciences^9,21^. In the model configuration, time-lagged responses were considered as well as different oceanic depth layers (as indicated above). The resulting models (for each region, depth layer, time-lag and timescale) are called the “sea-level *proxies*”. To further understand the features of the proxies, and analyze their performance, the following measures were used: the root mean square error (RMSE), which was normalized by the difference between the maximum and minimum value, and the explained variance (EV), i.e., the coefficient of determination (${R}^{2}$) (see Materials and Methods).

Based on the outcome metrics (Fig. S3), it was found that the depth average temperature over the top 100 m (i.e. MTA 100 m) was enough to characterize sea level variability changes in the regions within tropical latitudes (e.g., E PO, W AO, SW1 AO, SE AO, W/NW IO). The east Atlantic domain (i.e., E AO) and the Indo-Pacific basin (from the west Pacific warm-pool to the north and east Indian Ocean, i.e., NW2 PO, W PO, E IO, N1/N2 IO) are the exception, as the heat uptake in these regions can extend up to 700 m depth^22,23^. In these cases, therefore, a temperature estimate that includes the upper 700 m (e.g., MTA 700 m or OHC 700 m) would be a more suitable choice to capture the thermal expansion information. It is also interesting to note that, in the subtropical regions, MTA/OHC 700 m usually works better in the northern hemisphere, and MTA 100 m in the southern sites. Regardless of the region, generally, results do not show any time lag between our estimates and the SLA, also with few exceptions. For example, in the west and southwest Indian Ocean (W and SW2 IO) and northeast Atlantic Ocean (NE1/2 AO) the highest performance is achieved when a time lag is considered. This means that, in these regions, sea level changes can be detected in advance.

**Figure 3:**
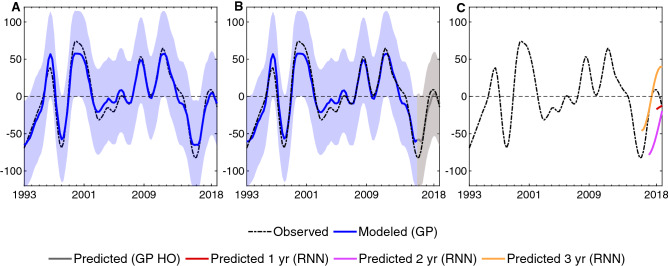
The regional modeled and predicted patterns are consistent with observed sea level variability. (**A**) Time series of the GP model (in blue) and observed sea level estimates (black) for the west Pacific Ocean region (shown in Fig. 2). (**B**) Same as in **A** but here the holdout method was used to predict sea level for the last 3 years of the record (in gray) by training the GP model from 1993 to 2015. (**C**) Sea level predictions for 1 (red), 2 (purple) and 3 (orange) years using the RNN method by training the observed sea level estimates (black) from 1993 to 2017, 2016 and 2015, respectively. The shaded envelops depict 95% prediction intervals. Sea level expressed in mm.

The time series shown in Fig. 3A correspond to the model obtained from one of the highest ranked temperature estimates (Fig. S3), compared against the observational-based sea level estimates for the west Pacific Ocean region (see Supplementary Information for the other regions). We chose this region for illustration purposes as it shows large sea level variations linked to oceanic mixed-layer heat content^8,24^. This region, which includes Indonesia's islands, is also among the locations most threatened by rising sea levels^1^. We can see, however, that overall the observed and modeled sea level estimates agree well in all regions influenced by low‐frequency climate variability, such as in the Indo-Pacific region (i.e., E IO, NW2 PO, W PO) and along the east Pacific coast (i.e., NE/E/SE PO). This also includes the western Indian (W IO) and central Atlantic oceans (W and E AO, extending into the NW2 AO and NE3 AO). In these regions, our GP model can explain at least 70% of the total variance, and correlation values range from 0.84 to 0.97 (see Fig. S7). In the other regions, the model performance differs between good (NE2 AO, NW IO, N2 IO, SE IO) and fairly good (SW PO, SW1 AO, N1 IO, NW1 AO) with correlation values higher than 0.80 and 0.70, respectively, excluding some regions where their climate conditions and local characteristics play a key role. The highest biases are found, for example, in the regions with mid-latitude wind-driven circulation, such as western boundary currents (e.g., SW2 IO, NW1 PO, SW2 and SW3 AO)^18^. Regions with high variance also coincide with places where salinity changes can be really large (as large as the thermosteric contribution, e.g., NE1 AO, SW1 IO or NW1 PO)^25^, or where there exists a combination of several effects (like in the SE AO)^26^. Even so, our model still offers a relatively good performance with regional correlation values always higher than 0.62. While this may vary at specific site locations (see Fig. 2), results suggest that the upper-thermal changes are still considerably impacting the regions and timescales studied here (Figs. S5-S8). Complementary analysis of other aspects (e.g., vertical land motion, tidal effects, storms, land ice change) could be performed to help better interpret results where relevant.

### Towards short-term predictions

We conducted further analysis using the GP model performed on a holdout set. This allows us to investigate, in particular, if by training the model using a certain percentage of the data, namely 88.5% (i.e., the 23 first years, from 1993 to 2015), we can construct the forecasting model with the remaining (11.5% of the) data (i.e., the last 3 years, from 2016 to 2018) (Materials and Methods). As seen in Fig. 3B and in Figs. S9-S12 (Supplementary Information), we found that, in general, the variability lies within the 95% prediction intervals. Therefore, our GP-based approach not only has the ability to identify regional coastal sea level variability changes from proxy data, but it could also be exploited in the future for filling data gaps (when information is missing or uncertain) and for proxy reconstructions of past sea level (1955–1993) changes. Nevertheless, even if the holdout method embeds a predictive response, it has its limitations when it comes to prediction of future changes as it uses a fraction of the full data for both training and predicting.

Hence, towards modeling and assessing sea-level predictions up to several years into the future, we implemented an alternative ML algorithm based on recurrent neural networks (RNNs), the long short-term memory (LSTM) (see Materials and Methods). This method has demonstrated excellent capabilities in learning real-world data that exhibits complex nonlinear patterns, especially, but not only, in the Earth sciences^11,27^. Unlike the GP method, the distinct advantage of the RNN method is that it can capture dynamic behavior and accounts for memory effects in the model’s architecture. This allows the model to perform forecasting beyond the in-sample training dataset without using additional data. To train the model we introduced a (training/validation) partition using the observed sea level data. We reserved a portion (the last years) of the historical record to test the robustness of predictions. Given the relatively limited length of the altimetry record, we only used one year for validation and up to three years for testing predictions. As expected, we observed that the model was often able to provide more accurate predictions for one to two years in advance, as opposed to three (see Figs. S13-S16 in Supplementary Information). However, in the particular case of the west Pacific Ocean, the rise in sea level was detected in all conducted experiments (Fig. 3C). It is also no surprise that, again, there are some regions (NW1 PO, SW1 and SW2 IO, SE AO, SW2 and SW3 AO) where sea levels are more difficult to predict, consistent with the above results. We believe, however, that the model could be improved by using more data when it becomes available. Noting that, in many cases, our model revealed correctly the magnitude of change (in relation to the 1993 baseline), even though our target was to simply detect the (positive/negative) tendency towards an increase/decrease in future regional sea levels.

Results show that both the GP and RNN models are promising ML techniques for automatically modeling and predicting short-term regional coastal sea level changes. Furthermore, the flexibility of the proposed framework to analyze multiple data enables the possibility to explore in the future other proxy variables or a combination of them.

## Conclusion

Machine learning-based strategies for predicting the nonlinear behavior of short-term regional coastal sea level changes are still rarely present. Our machine learning approach has shed new light on this problem in two ways. One on hand, it has allowed to mimic sea level variability changes to a large extent in many coastal regions around the Pacific, Indian and Atlantic oceans, by establishing physical relationships between input temperature variables from the upper layers of open ocean regions (that play a decisive role in regional “thermosteric” expansion) and sea level estimates at the coastal sites of these regions. Beyond that, it has been possible to provide reasonably accurate region-specific predictions of the sea level tendency for one to two/three years. Thereby, the introduced methodology offers a view that is not purely statistical, and also fills the gap between the coastal adaptation planners demand and the existing multidecadal-to-century projections of sea level change.

## Materials and methods

### Observational datasets

Two sources of observational-based data were analyzed: (i) daily satellite mean sea level anomalies (SLA) through 2019 (https://resources.marine.copernicus.eu/?option=com_csw&view=details&product_id=SEALEVEL_GLO_PHY_L4_REP_OBSERVATIONS_008_047), resampled at seasonal (3-month) resolution; (ii) seasonal in-situ-based vertically-averaged temperature anomalies for the 0–100 m and 0–700 m layers (MTA 100 m, MTA 700 m) and heat content for the 0–700 m layer (OHC 700 m) through present (https://www.nodc.noaa.gov/OC5/3M_HEAT_CONTENT/). These temperature-based estimates have been demonstrated to have good quality through direct comparisons with field measurements and intercomparisons with other satellite-derived products^28^. All products have been expressed from their 1993–2018 mean and smoothed with a 1-, 3-, and 5-year *Savitzky-Golay* filter^29^ (that smooths according to a quadratic polynomial that is fitted over each window) to help distinguish natural internal fluctuations from the historical anthropogenic warming signal and assess multi-time-scale variability. To get the regional estimates, instead of estimating the area-weighted average over each oceanic region (shown in Fig. 2), we chose the median value^30^, as it has a slightly better regional representation, especially where the noise of interannual variability is high, as we have seen here.

### Model experiments and evaluation

For our modelling and prediction experiments, two machine learning techniques were used; a Gaussian process (GP) regression model and a recurrent neural network (RNN) with long-short term memory (LSTM) units. The GPs are state-of-the-art non-parametric methods (and the preferred choice) for machine learning regression, while RNNs apply recurrent operations to data in a neural-network fashion to account for and forecast complex nonlinear dynamics.

The GP methods were used in two different ways. First, to generate the GP regression model, we trained the different temperature input variables with the satellite altimetry sea level (1993–2018) record using the MATLAB *fitrgp* function (with *KernelFunction* = *Exponential, FitMethod* = *None, PredictMethod* = *Exact*, as the model specifications^21^). A lag of up to 15 months for the temperature input variables was selected following a series of different time lags to explore a lagged-response between the temperatures and sea level estimates. As the GP models are trained, we compute the mean squared error (MSE) to assess which input variables generally performs best on each region. The coefficient of determination was also estimated to account for goodness-of-fit: ${R}^{2}=1-{\sum }_{i=1}^{N}{({y}_{i}-{y}_{i}^{*})}^{2}/{\sum }_{i=1}^{N}{({y}_{i}-{\bar{y}})}^{2}$, where ${y}_{i}$ and ${y}_{i}^{*}$ are the observed sea level estimate and the model prediction from the temperature data, respectively, for each region/location. Note that the right side of the equation represents what the model cannot explain, hence ${R}^{2}$ (or explained variance, EV) should be interpreted as the proportion of variability that is explained by the model. $R$, its square-root value, is equivalent to a measure of correlation, indicating how strongly related the coupled patterns are. According to this performance criterion, the variables are categorized in terms of rankings.

Alternatively, and the second use of GPs, the same sort of analysis could be performed on a holdout set to test the predictive performance of the GP method using the highest ranked temperature estimates. With the hold-out approach, we use a relatively high percentage of the (temperature and sea level) data for training a new model (again using the *fitrgp* function) and this model with the rest of the (temperature) data to predict outcomes (using the MATLAB *predict* tool). In this study, the partitioning of the dataset is not random but done into consecutive periods of time to respect a certain date. We use the same metrics (EV and R) to assess robustness of the model outputs.

An additional experiment was performed to explore the potential use of LSTMs/RNNs techniques to predict future sea-level tendencies. These techniques provide the possibility to take a set of data and train a model which can be used to generate reasonable predictions without using another set of data to model from ^11^. In this analysis, the RNN is directly trained against the sea level data using the MATLAB *trainNetwork* tool with the below-specified training options and Long Short-Term Memory (LSTM) network architecture^27^. The predicted responses were obtained by applying the MATLAB *predictAndUpdateState* function with the trained recurrent neural network. This function also updates the network state at each prediction, giving it memory of previous inputs. All hyper-parameters in the network were determined via cross-validation in the training set. The best overall performance was obtained with the ‘*adam’* solver (a good optimization algorithm for training a neural network) and 4 layers with 2–20 hidden units. The hidden units allow the networks to produce a nonlinear response to the inputs and represent “mean” predicted output. It was observed that fewer hidden units did not fully learn the complexity of the patterns, and that additional hidden units could lead to overfitting the training dataset. The number of epochs (i.e., the number of complete passes through the training set) is automatically set by the above functions based on the loss calculation, so that the network will stop training when the loss on the validation set has been equal to (or larger than) the previous smallest loss for several consecutive epochs, and it differs among regions. The RMSE loss of each model run is hence used as the performance indicator for each iteration. For this experiment, training datasets were split for training, validation and testing.

## Supplementary Information

Supplementary Figures.
