# Supplementary material for: Predicting regional coastal sea level changes with machine learning
Source: Sci Rep. 2021 Apr 7;11:7650. doi: 10.1038/s41598-021-87460-z (PMC8027398; doi:10.1038/s41598-021-87460-z)
Supplement: Supplementary file 1 — Supplementary Figures. [file 41598_2021_87460_MOESM1_ESM.pdf]

Supplementary Information for

Predicting regional coastal sea level changes with machine learning

Veronica Nieves[1*], Cristina Radin[1], and Gustau Camps-Valls[1]

[1]Image Processing Laboratory, University of Valencia, Valencia, Spain

[*]e-mail: veronica.nieves@uv.es

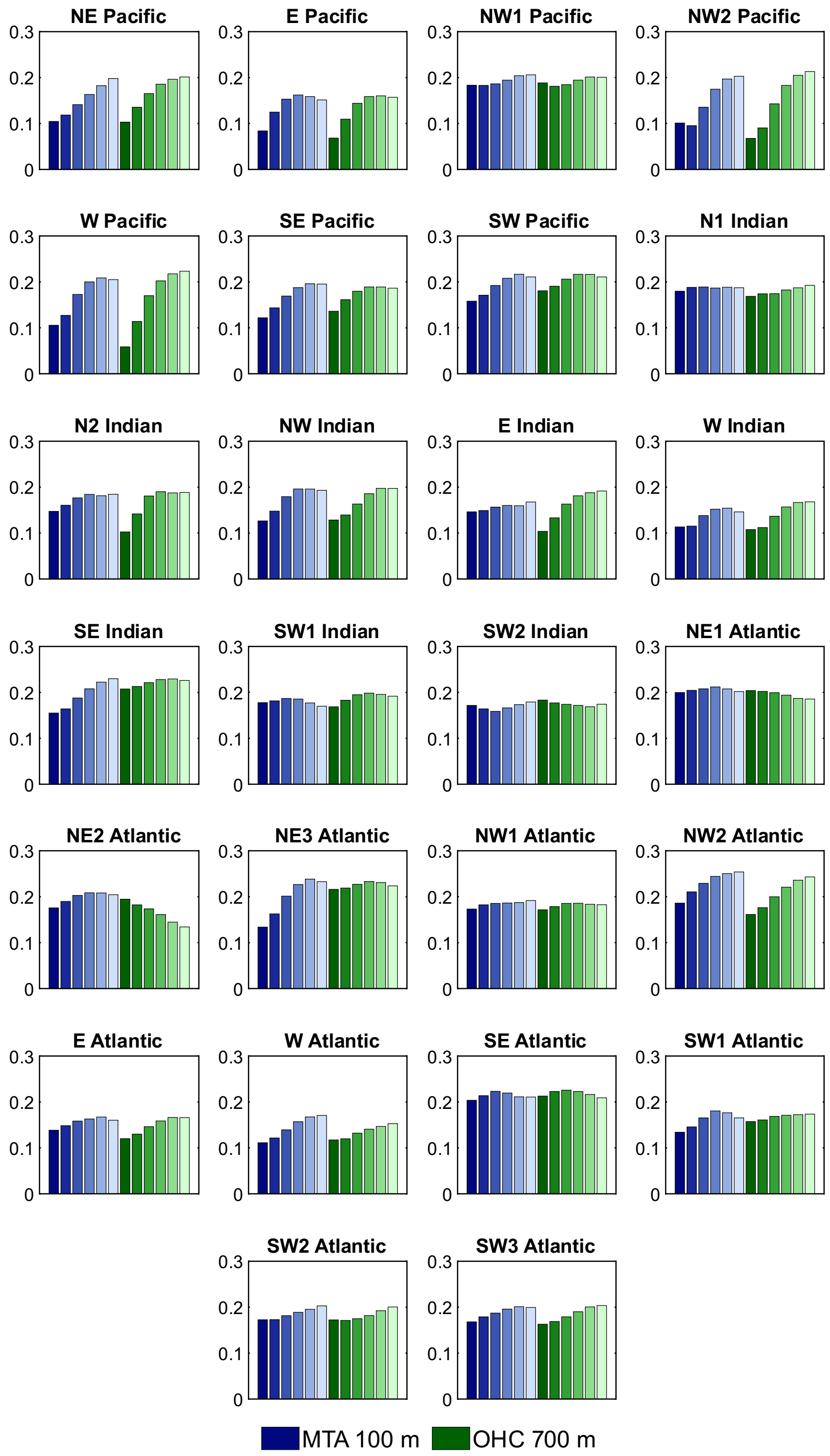

Fig. S1. Normalized RMSE (NRMSE) between the GP model generated from the temperature-based estimates (MTA 100 m in blue and OHC 700 m in green) and observed sea level estimates for each region (in Figure 2) and time lag. Time lags from 0 (dark) to 5 (light color) correspond to 0, 3, 6, 9, 12, and 15 months, respectively. All estimates were detrended over the period 1993 to 2018.

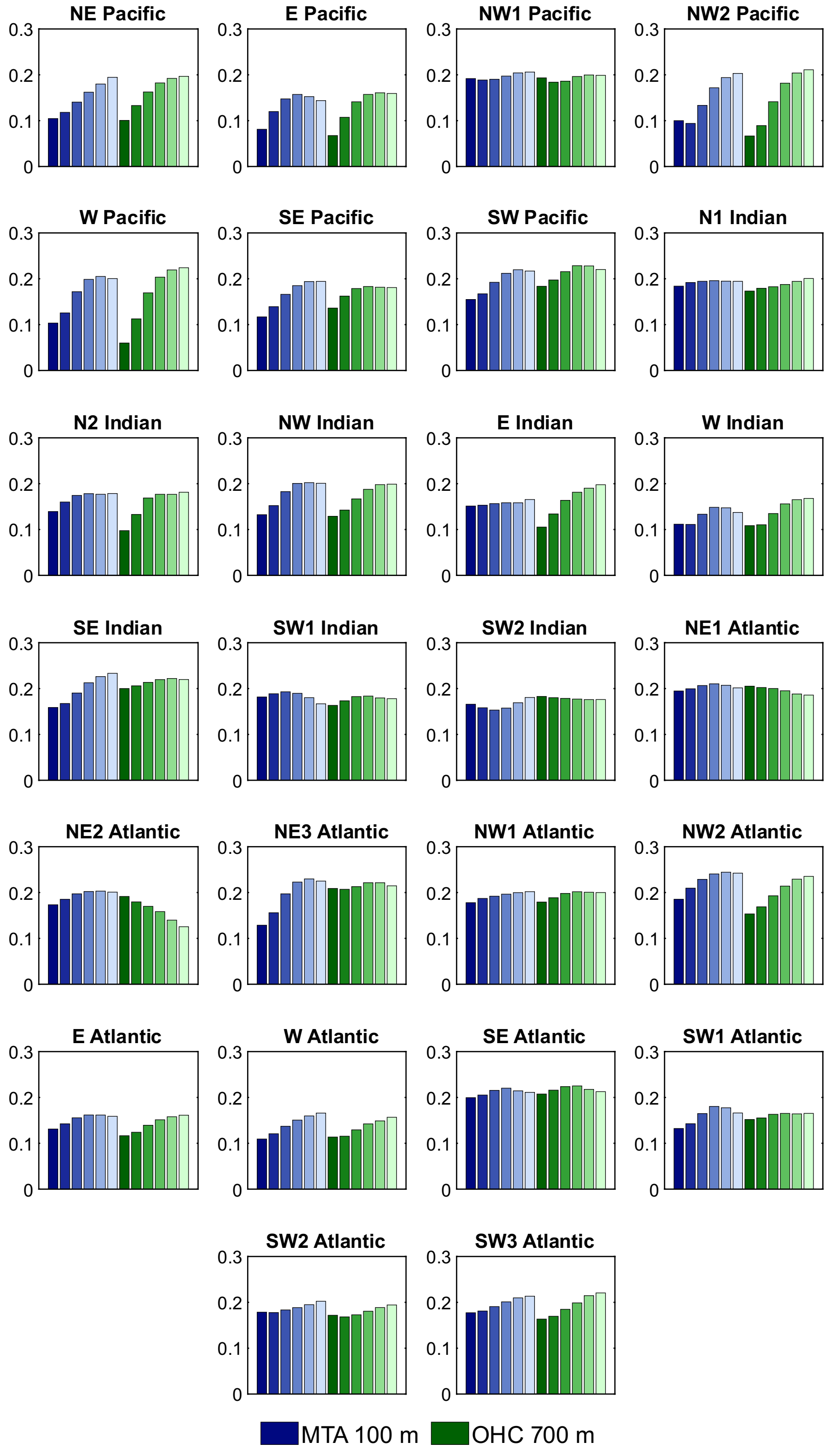

Fig. S2**.** As in Figure S1, but here data was smoothed with a 1-year filter.

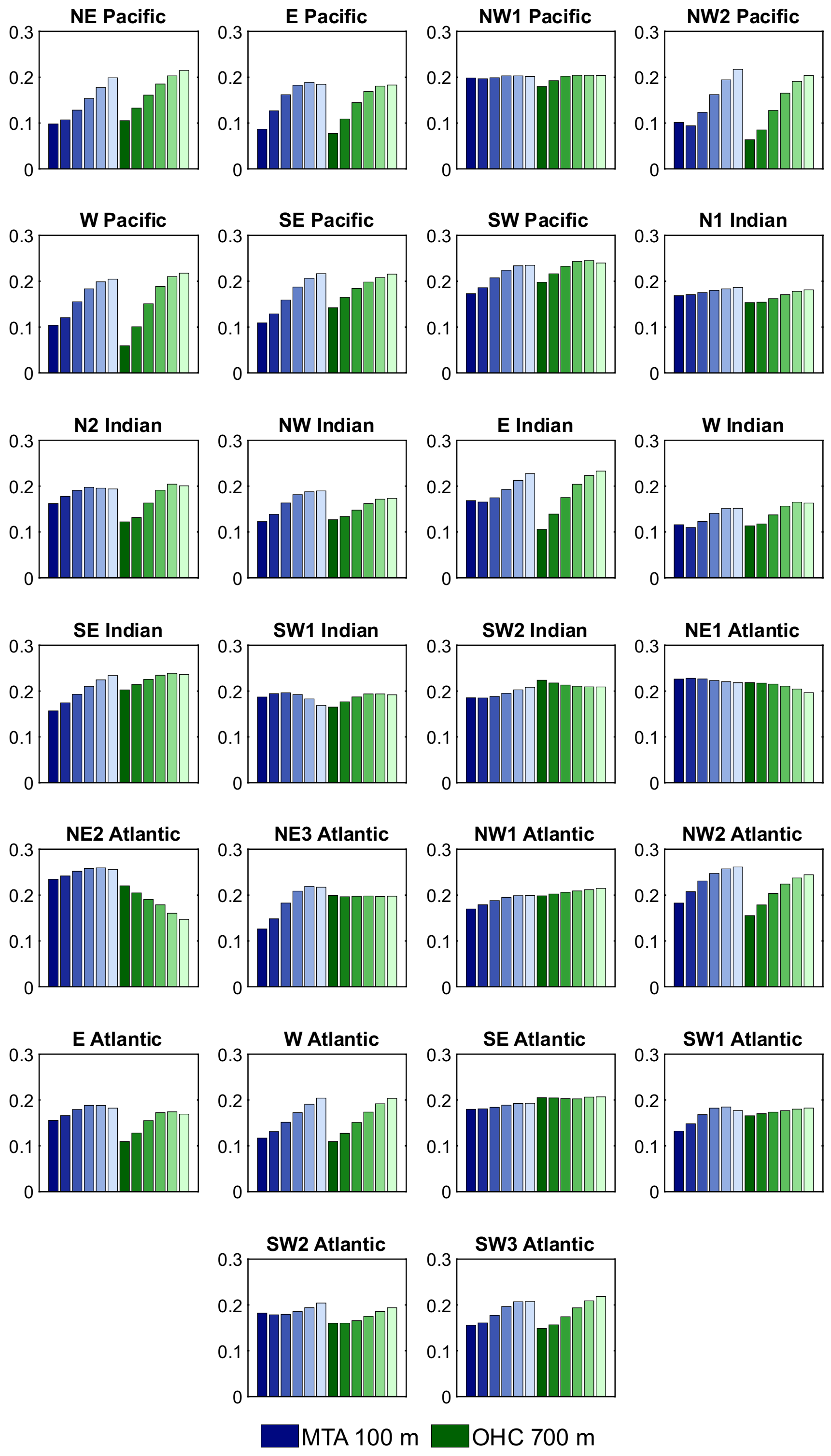

Fig. S3. As in Figure S1, but here data was smoothed with a 3-year filter.

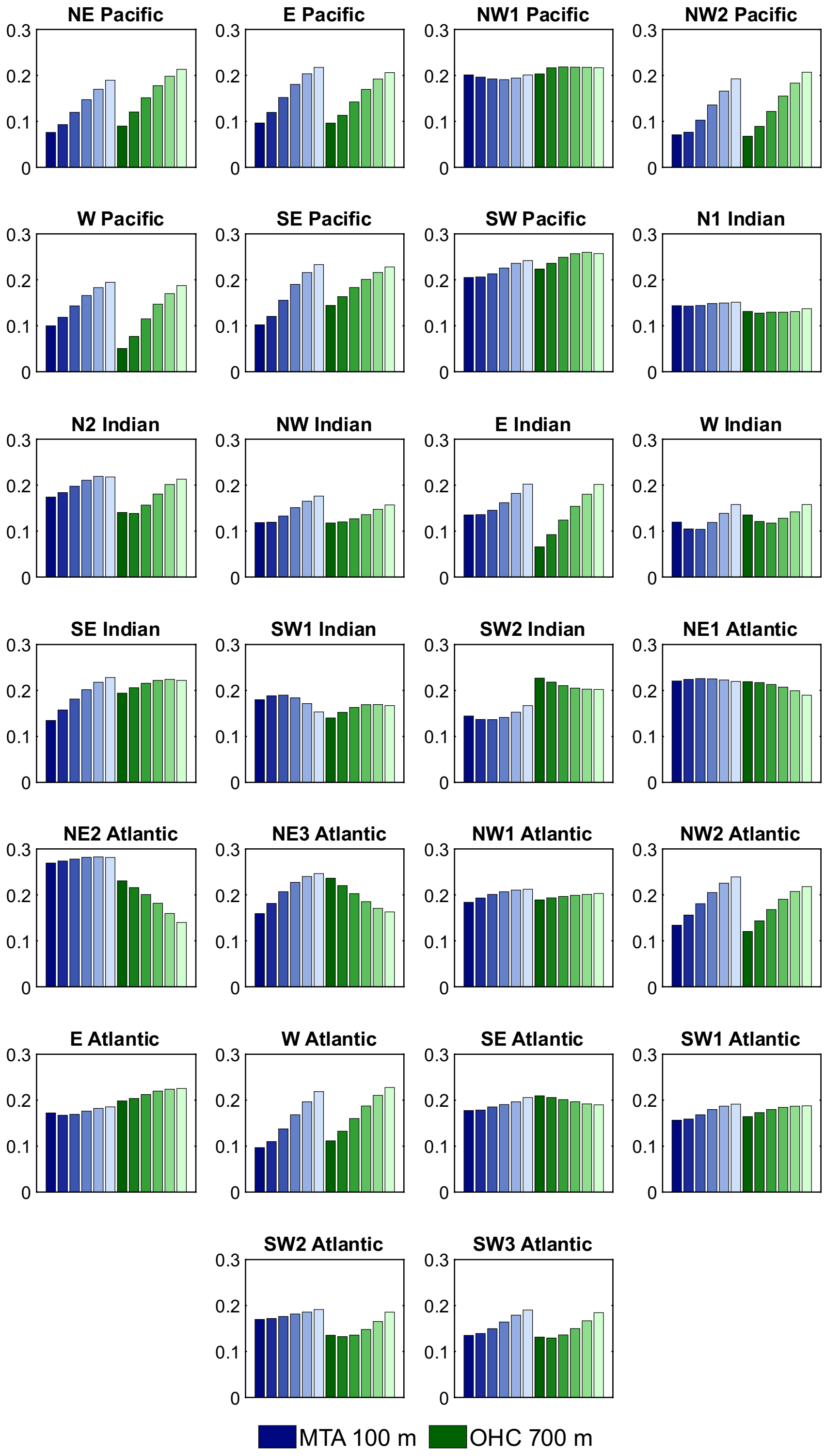

Fig. S4. As in Figure S1, but here data was smoothed with a 5-year filter.

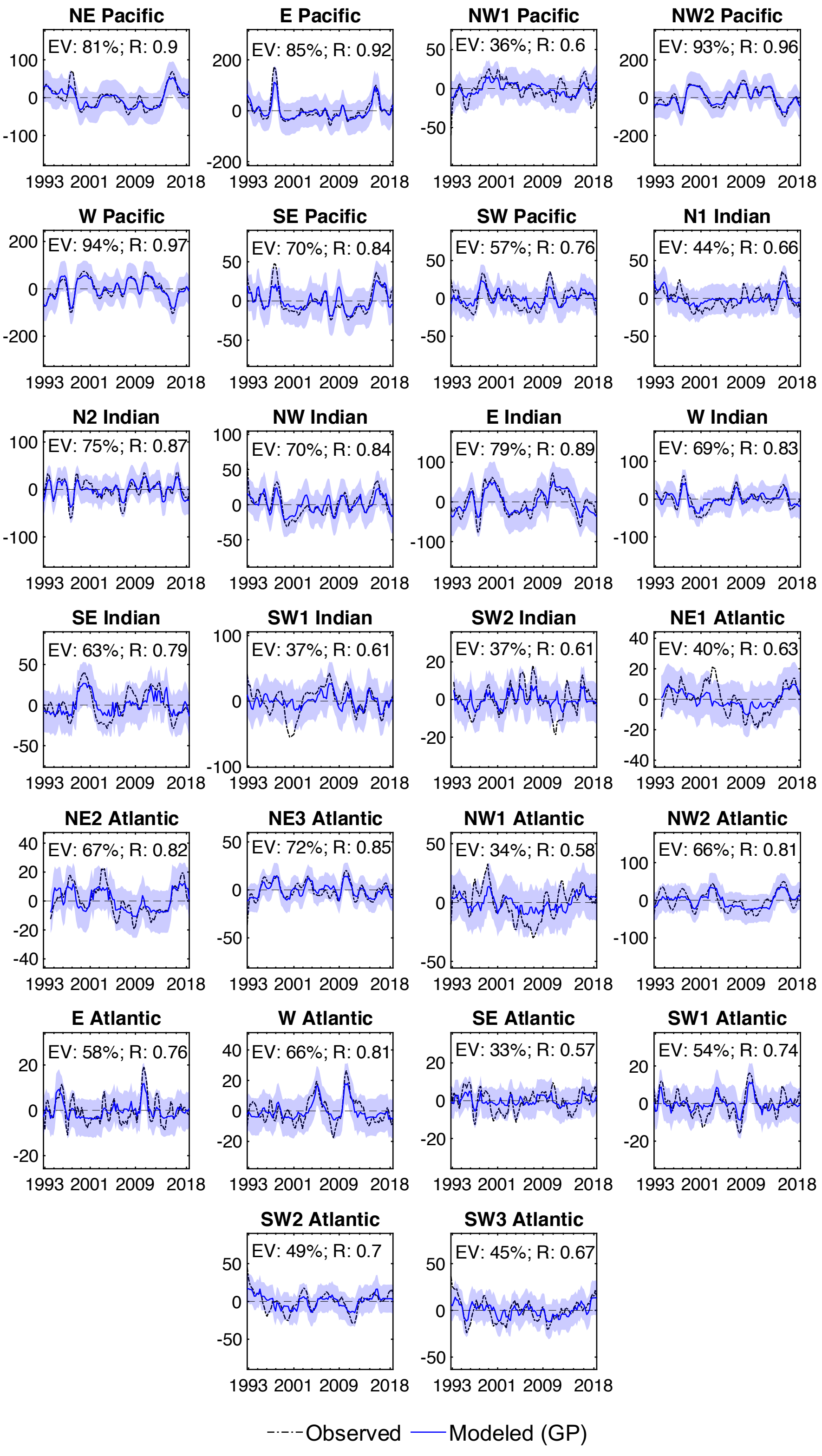

Fig. S5. Time series of the GP model (in blue) and observed sea level estimates (black) for all regions. The shaded envelops depict 95% prediction intervals. Sea level expressed in mm. EV and R are the explained variance and coefficient of determination (see Methods).

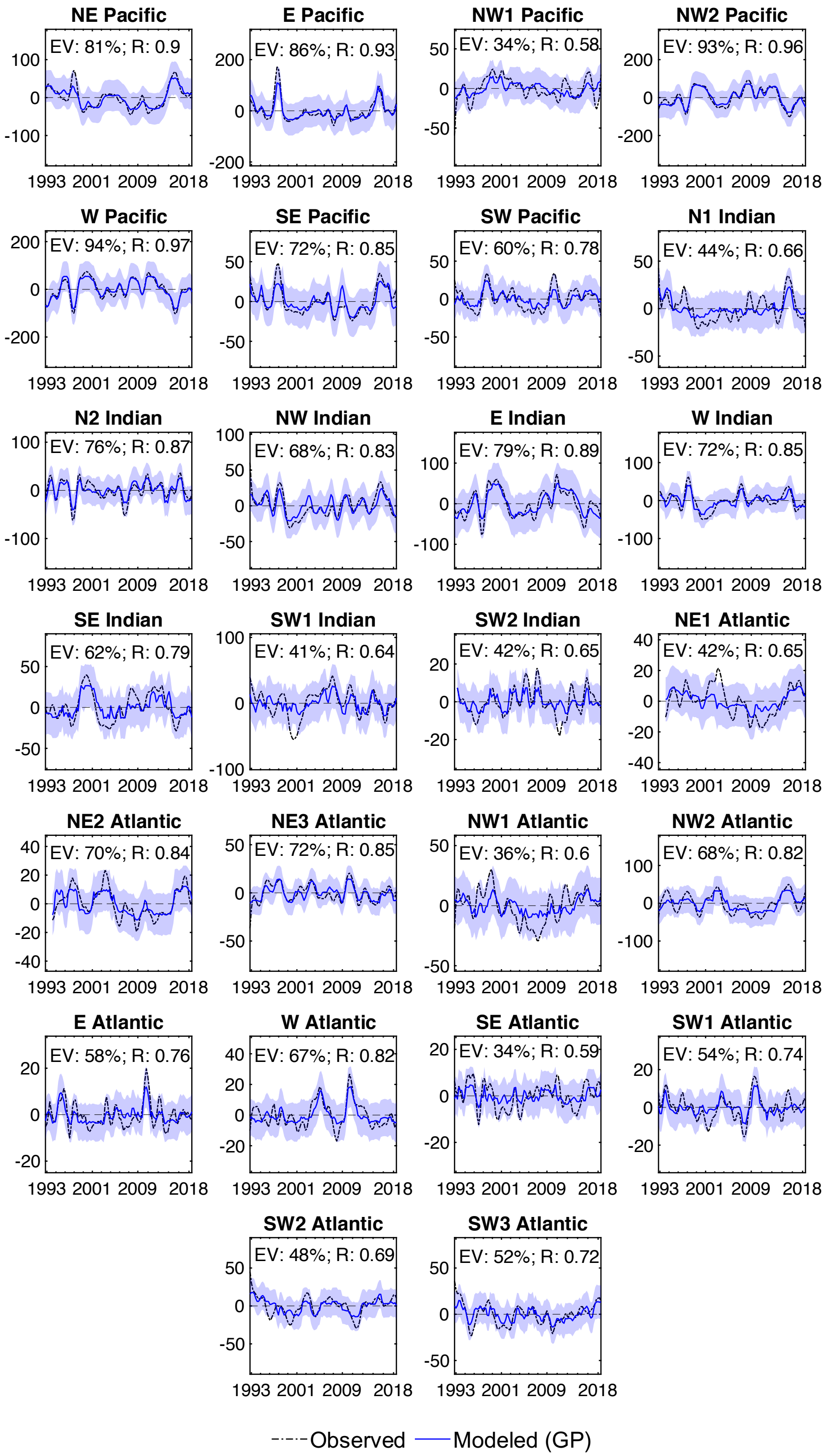

Fig. S6. As in Figure S5, but here data was smoothed with a 1-year filter.

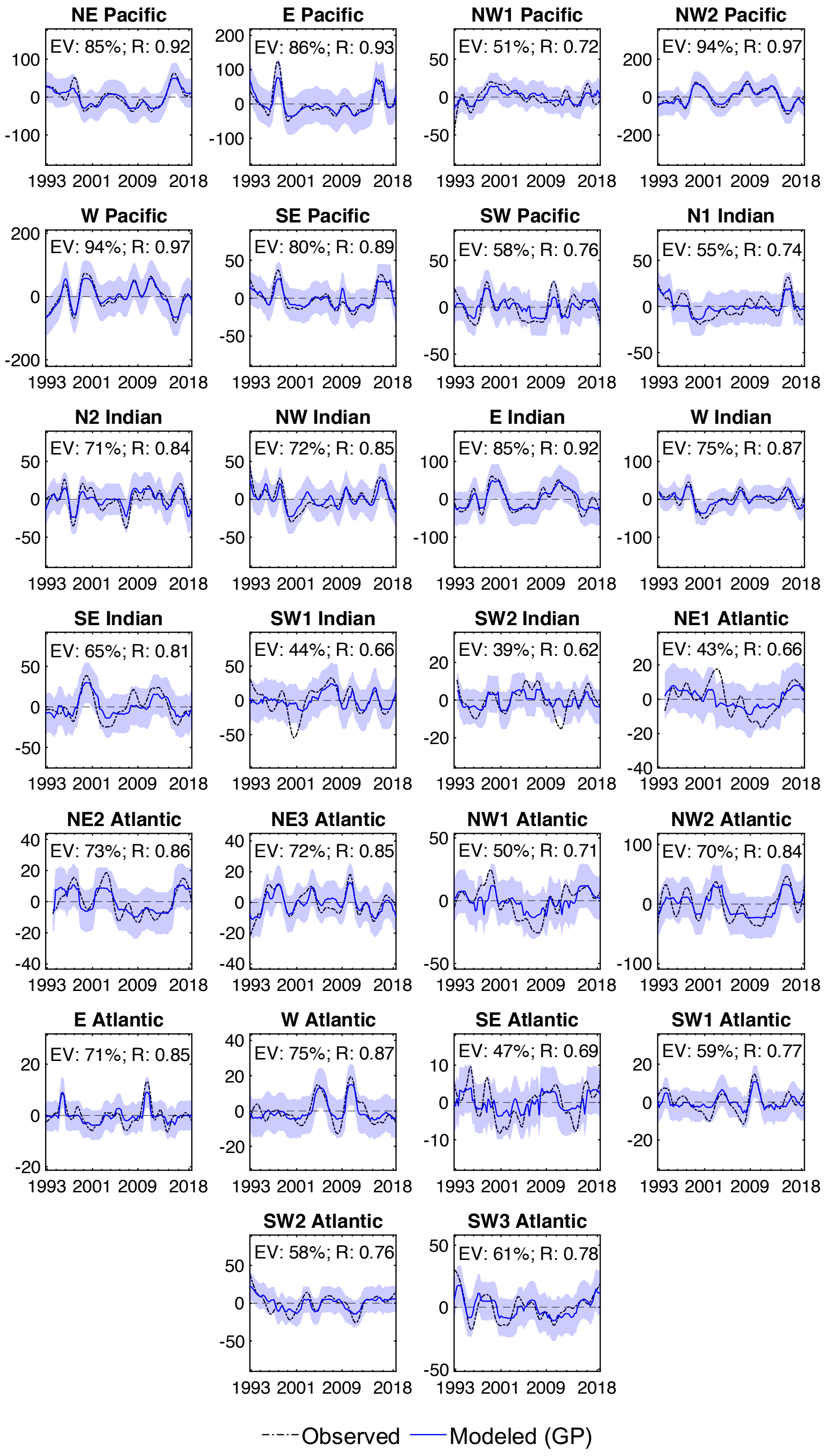

Fig. S7**.** As in Figure S5, but here data was smoothed with a 3-year filter.

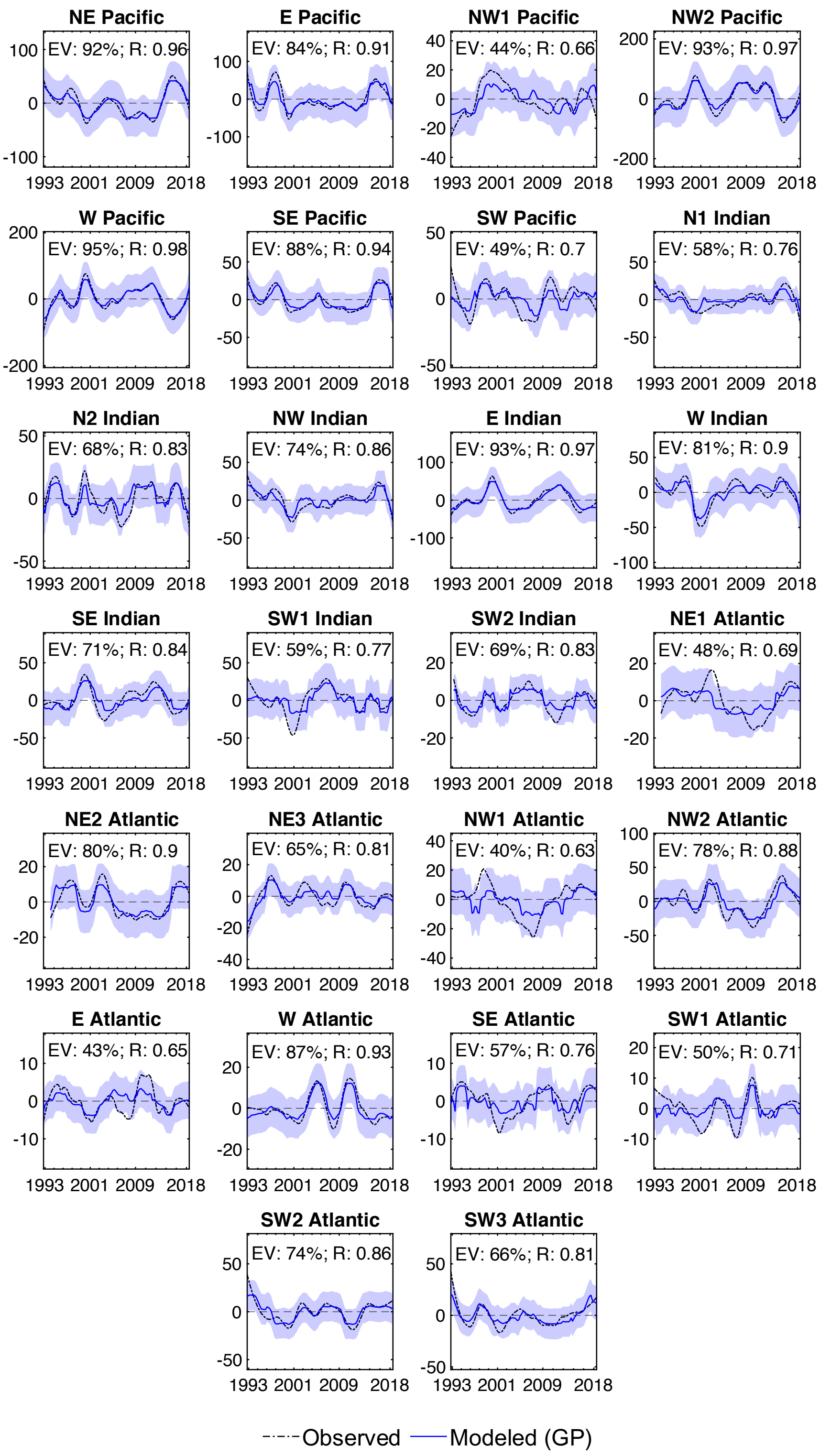

Fig. S8. As in Figure S5, but here data was smoothed with a 5-year filter.

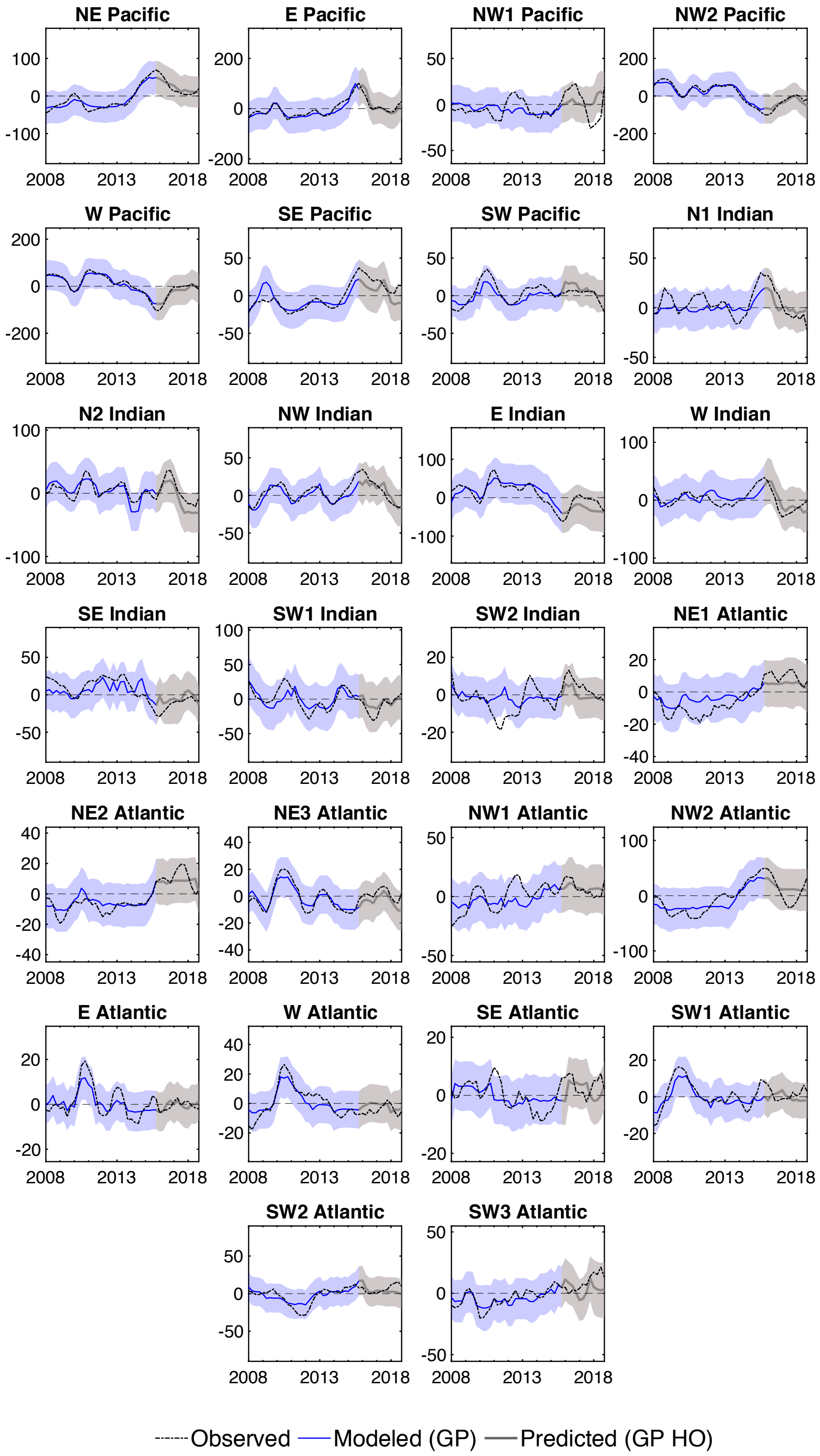

Fig. S9. Same as in Figure S5 but here the holdout method was used to predict sea level for the last 3 years of the record (in gray) by training the GP model from 1993 to 2015. For clarity, results are only shown from the year 2008 onward.

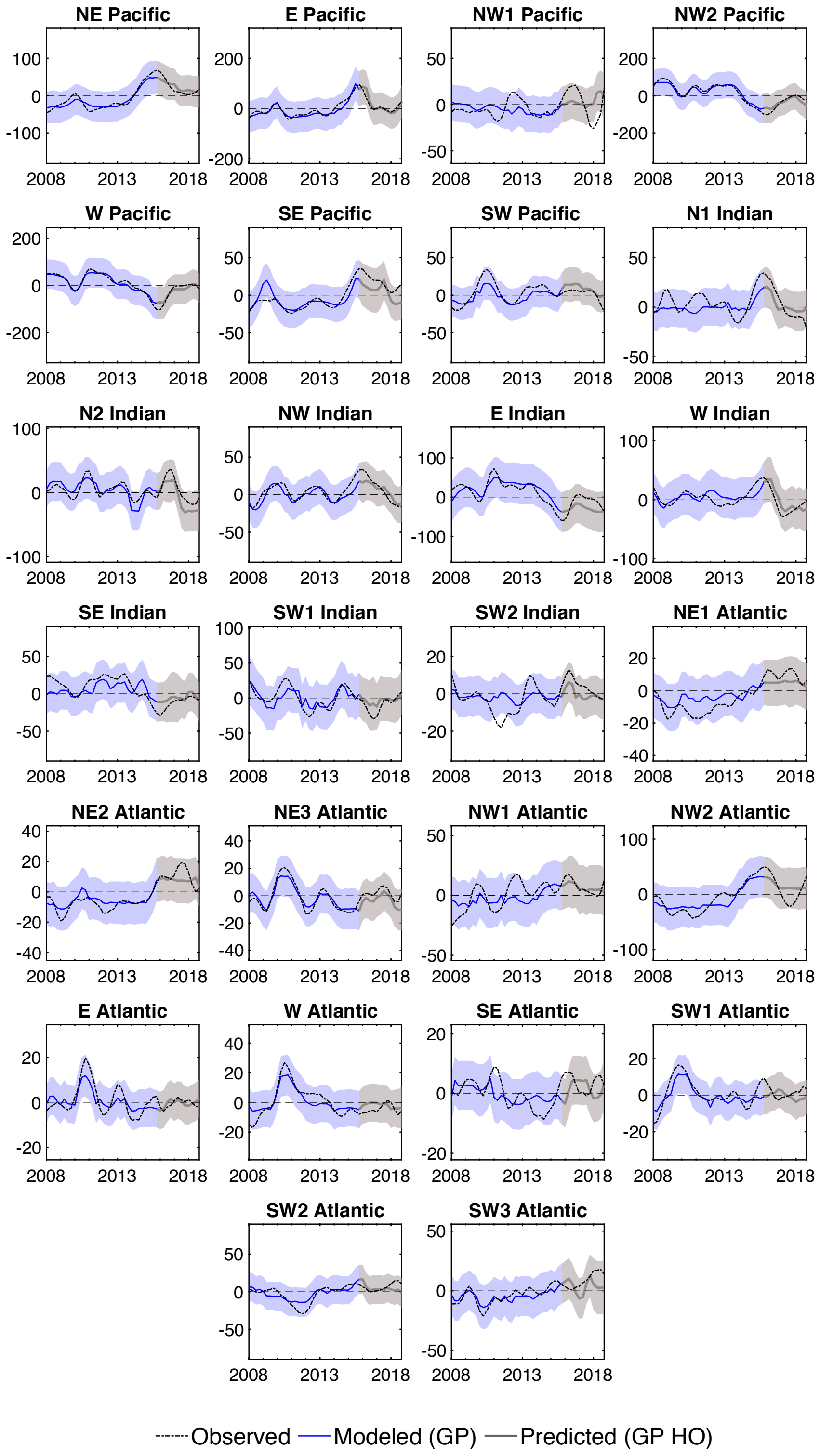

Fig. S10. As in Figure S9, but here data was smoothed with a 1-year filter.

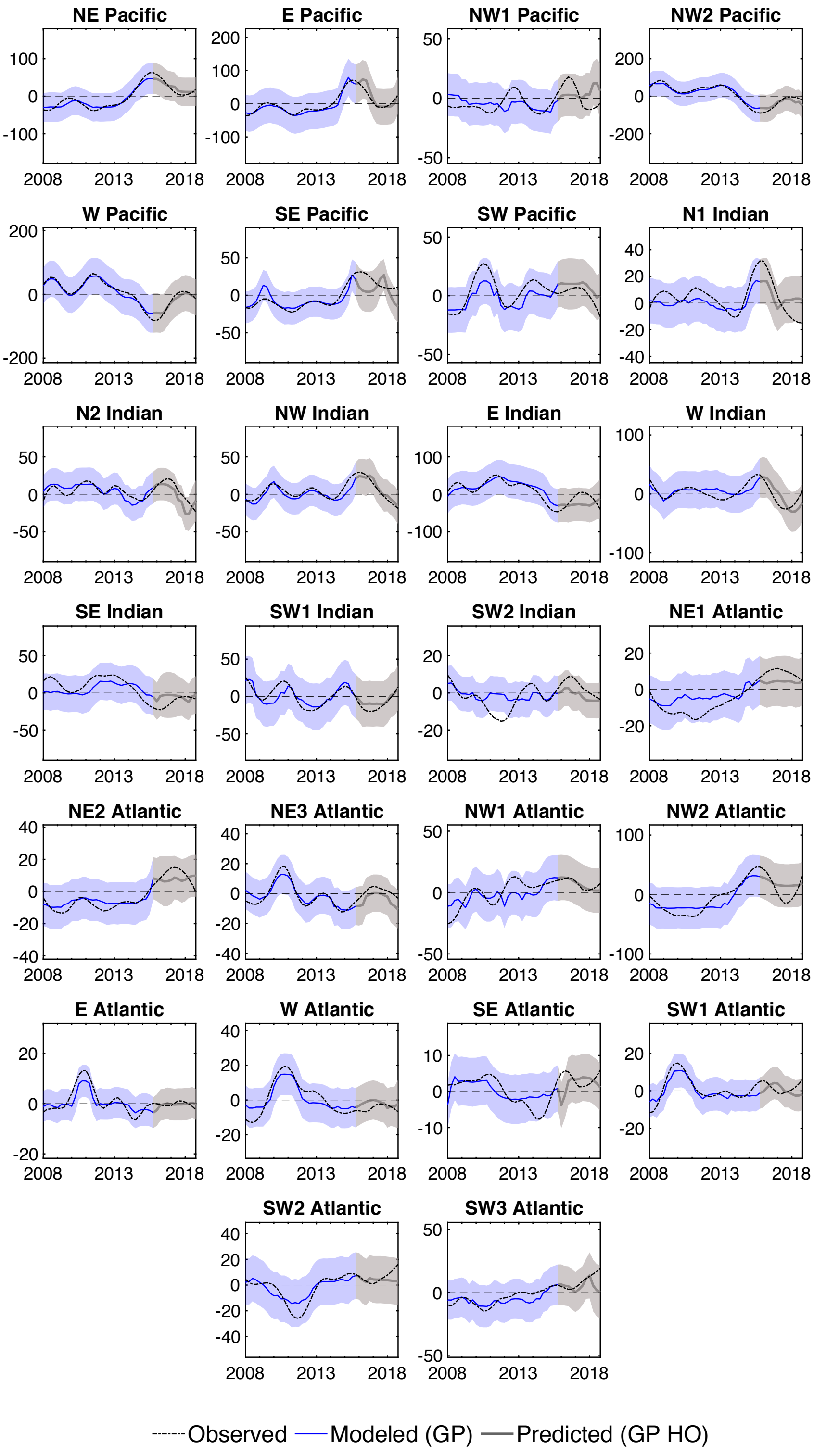

Fig. S11. As in Figure S9, but here data was smoothed with a 3-year filter.

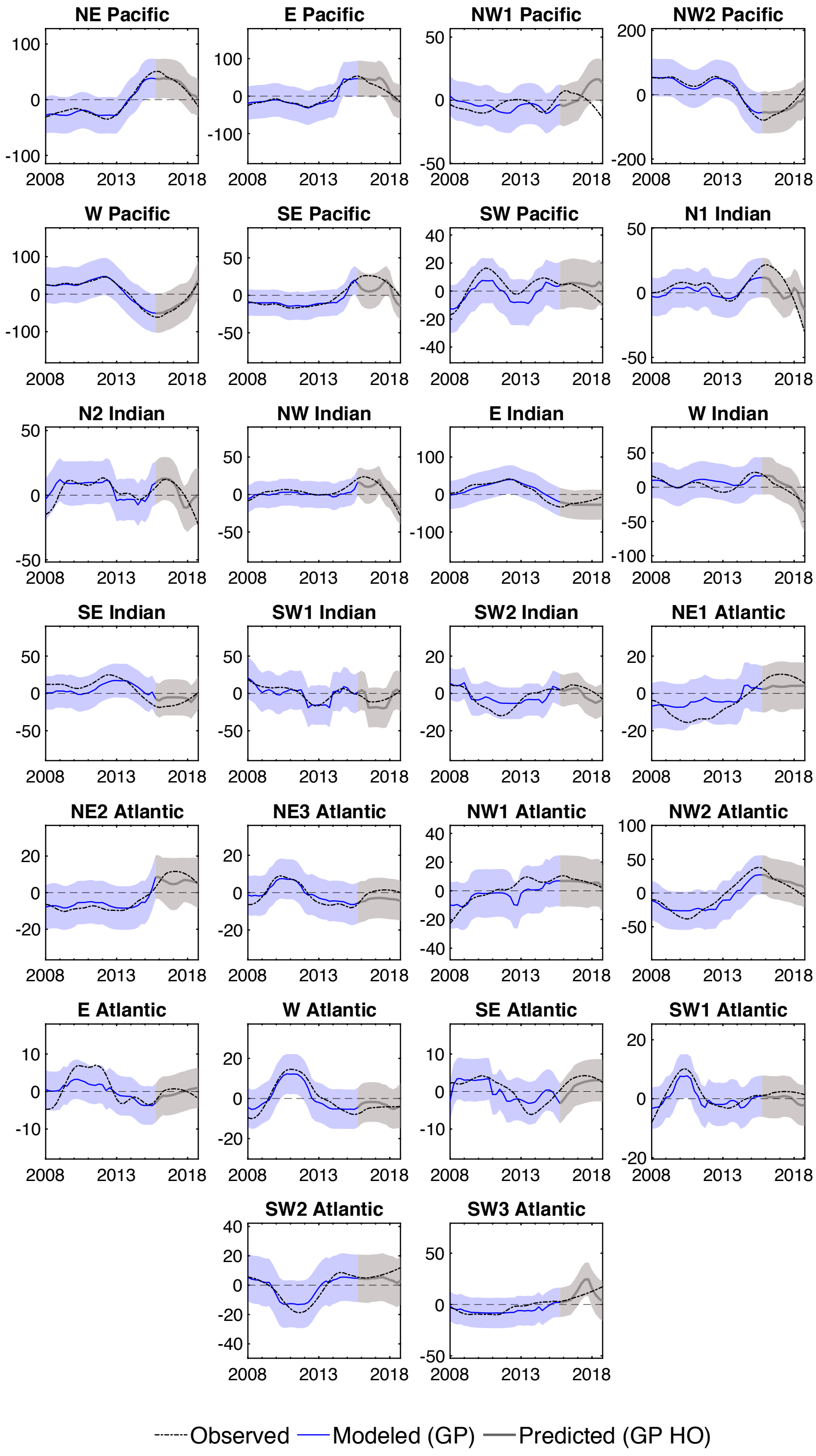

Fig. S12. As in Figure S9, but here data was smoothed with a 5-year filter.

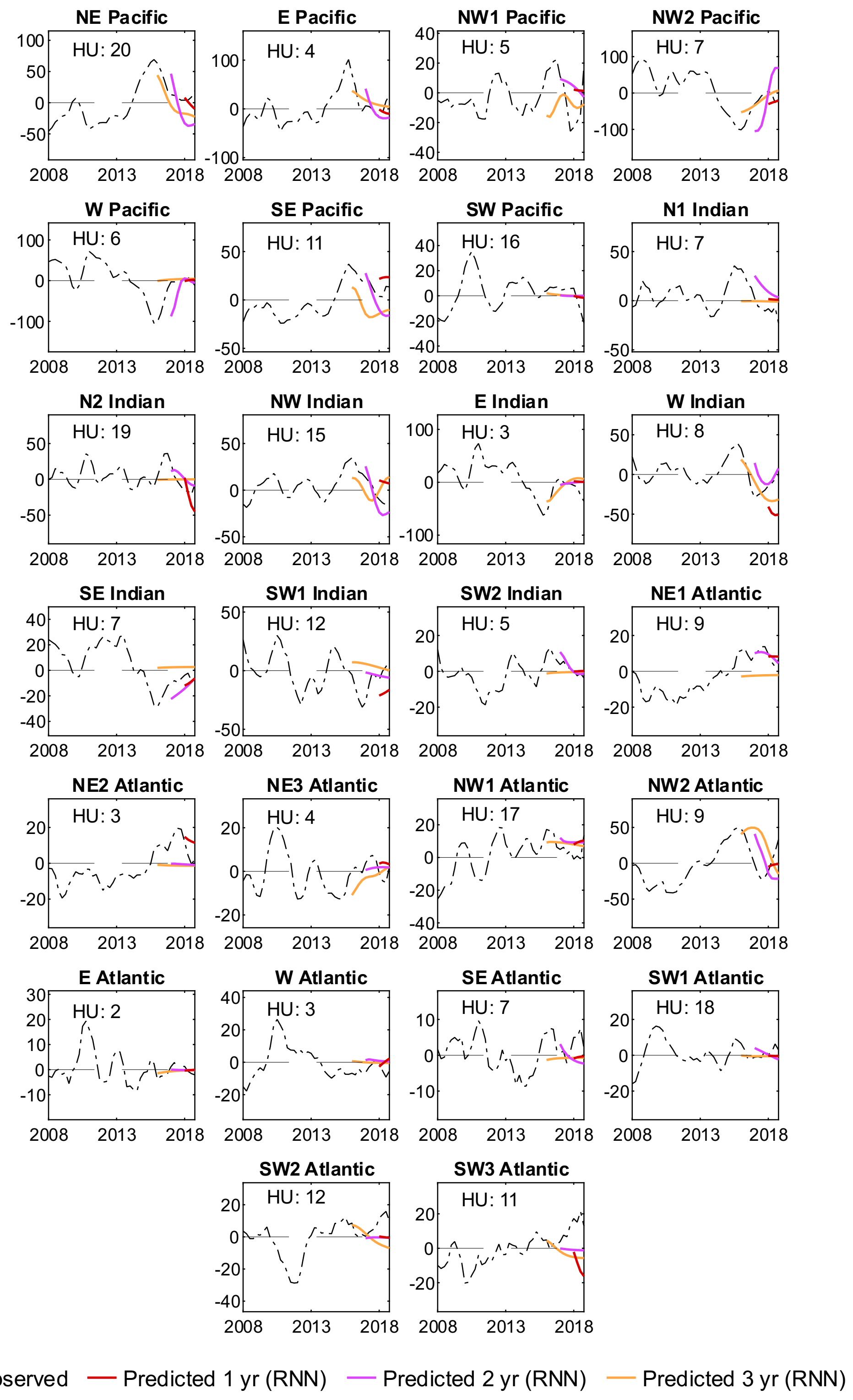

Fig. S13. Sea level predictions for 1 (red), 2 (purple) and 3 (orange) years using the RNN method by training the observed sea level estimates (black) from 1993 to 2017, 2016 and 2015, respectively. For clarity, results are only shown from the year 2008 onward. HU stands for hidden units (see Methods).

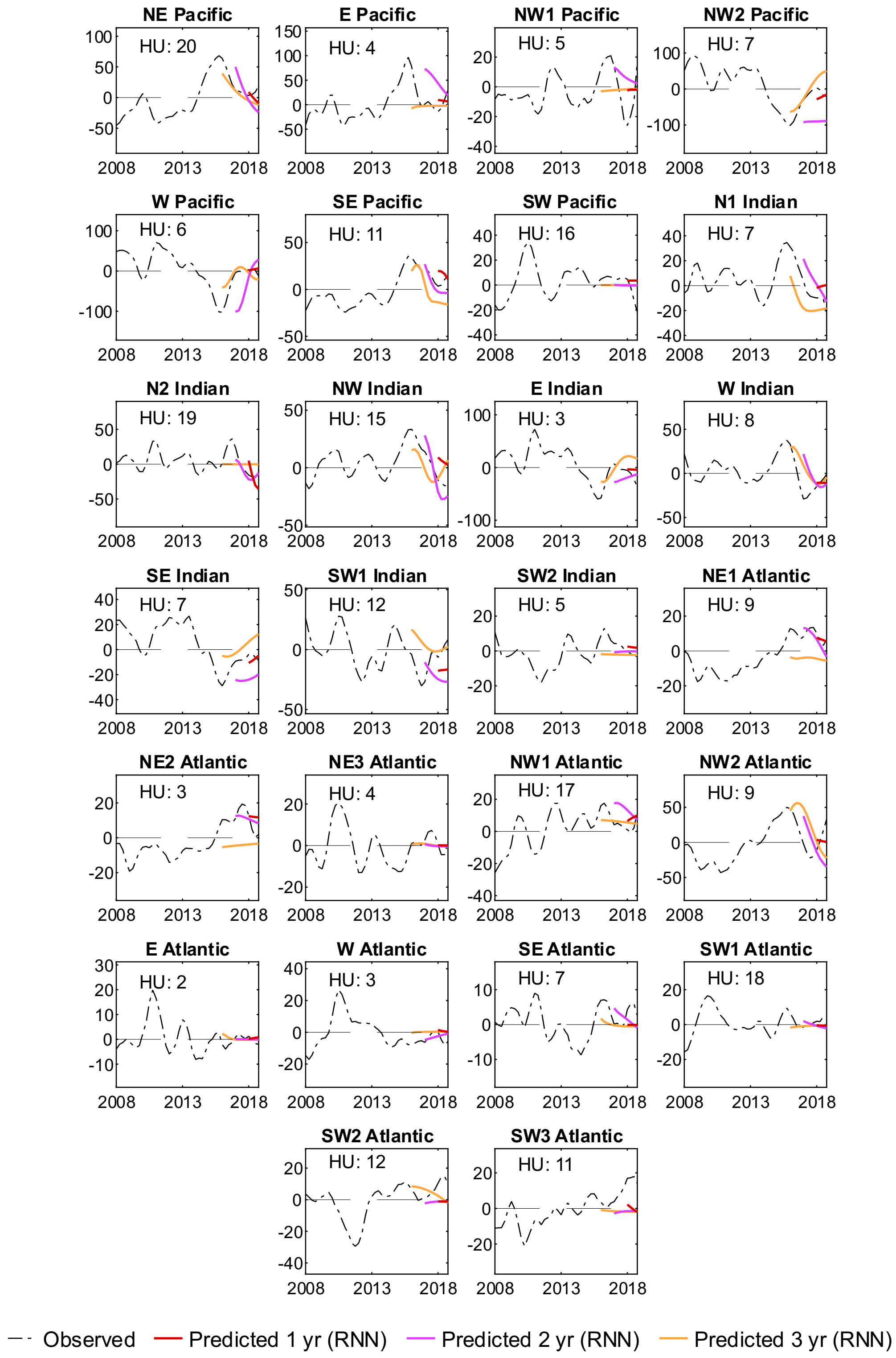

Fig. S14. As in Figure S13, but here data was smoothed with a 1-year filter.

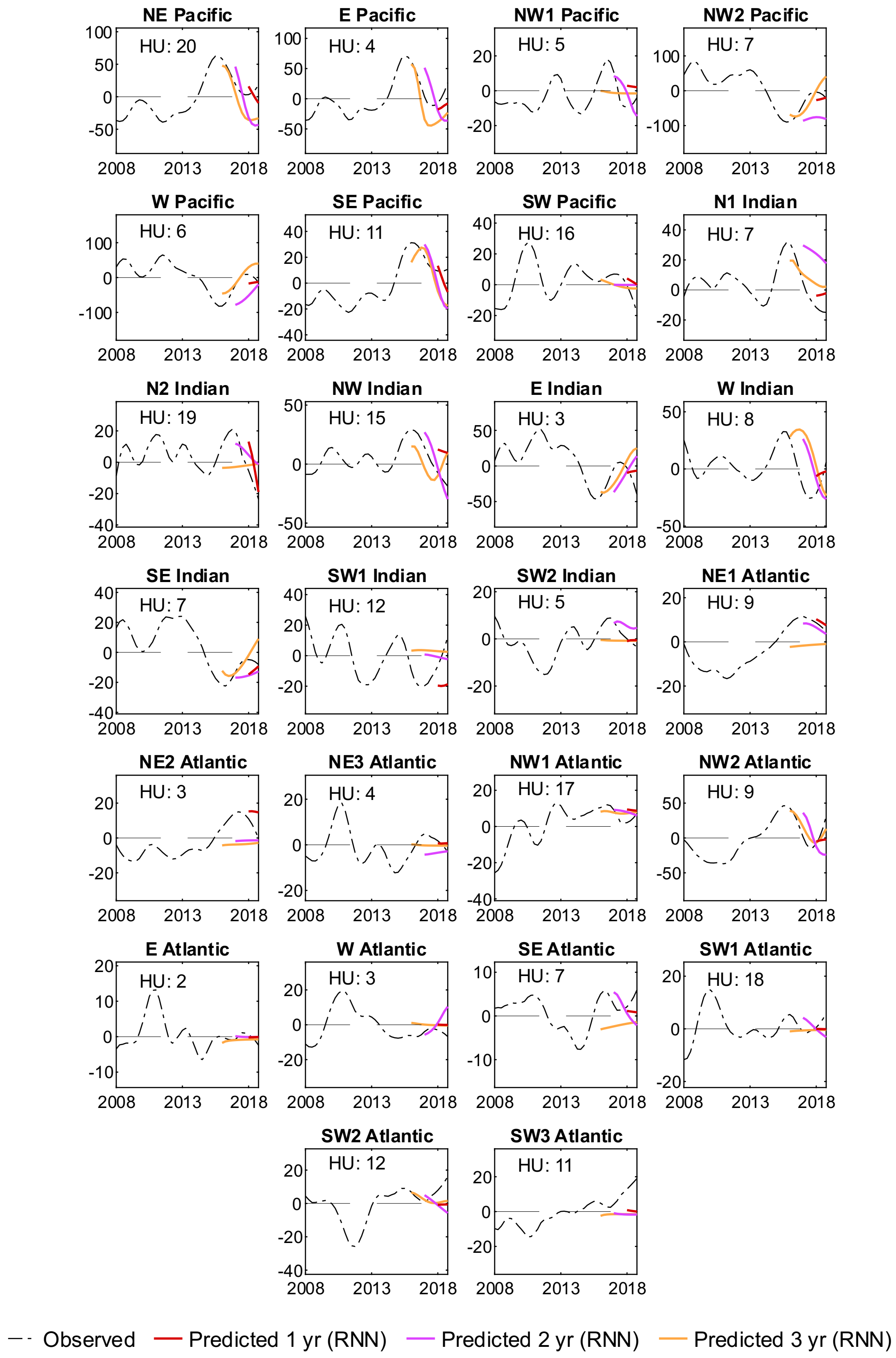

Fig. S15. As in Figure S13, but here data was smoothed with a 3-year filter.

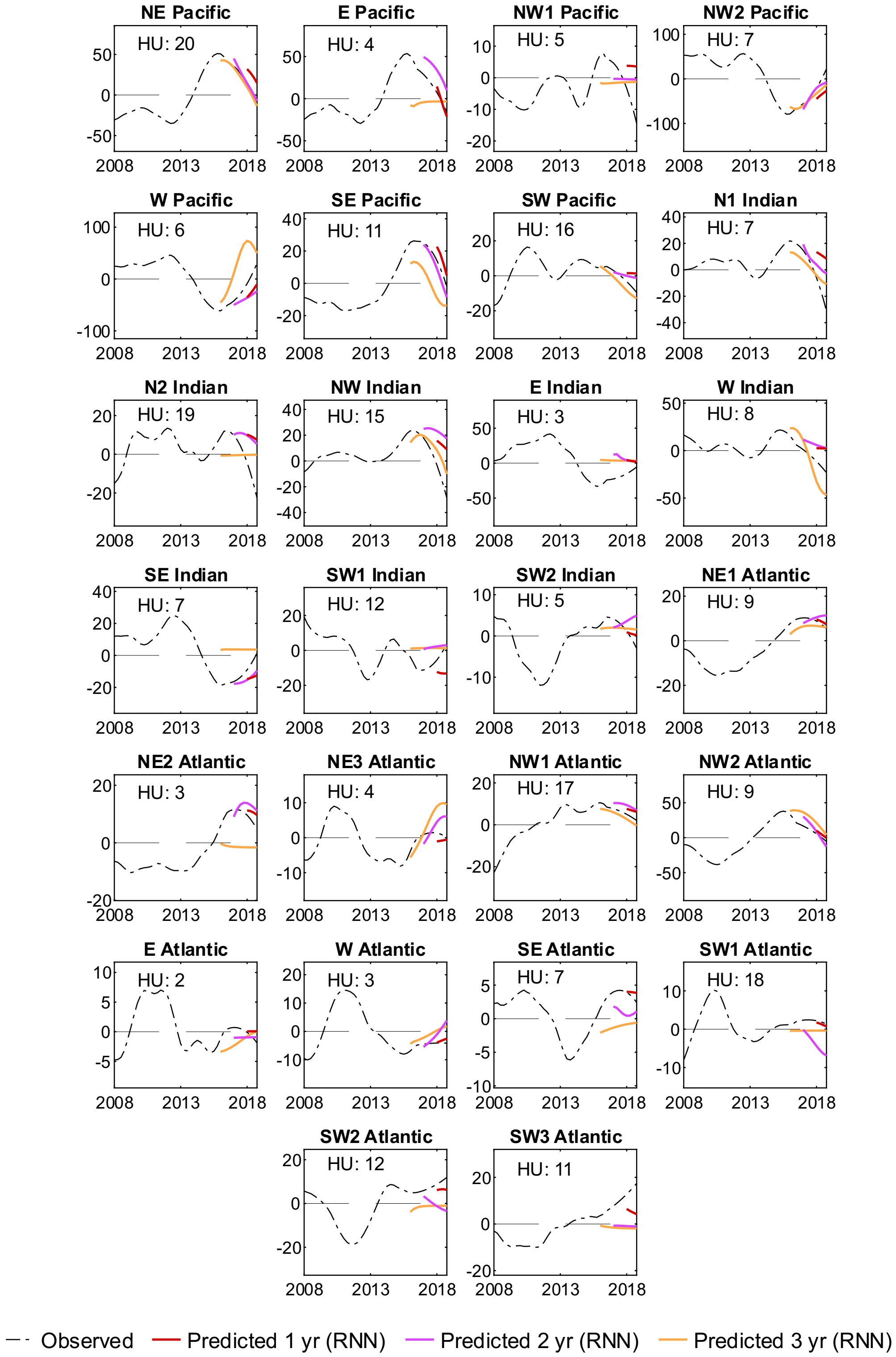

Fig. S16. As in Figure S13, but here data was smoothed with a 5-year filter.
